# Visual Field Deficits in Albinism in Comparison to Idiopathic Infantile Nystagmus

**DOI:** 10.1167/iovs.65.2.13

**Published:** 2024-02-06

**Authors:** Viral Sheth, Rebecca J. McLean, Zhanhan Tu, Sarim Ather, Irene Gottlob, Frank A. Proudlock

**Affiliations:** 1 Health Sciences School, University of Sheffield, Sheffield, Yorkshire, United Kingdom; 2 The University of Leicester Ulverscroft Eye Unit, Psychology and Vision Sciences, University of Leicester, Robert Kilpatrick Clinical Sciences Building, Leicester Royal Infirmary, Leicester, United Kingdom; 3 Oxford University Hospitals NHS Foundation Trust, Headley Way, Headington, Oxfordshire, United Kingdom; 4 Department of Neurology, Cooper University Health Care, Cooper Medical School of Rowan University, Camden, New Jersey, United States

**Keywords:** visual fields, albinism, nystagmus, visual acuity, optical coherence tomography

## Abstract

**Purpose:**

This is the first systematic comparison of visual field (VF) deficits in people with albinism (PwA) and idiopathic infantile nystagmus (PwIIN) using static perimetry. We also compare best-corrected visual acuity (BCVA) and optical coherence tomography measures of the fovea, parafovea, and circumpapillary retinal nerve fiber layer in PwA.

**Methods:**

VF testing was performed on 62 PwA and 36 PwIIN using a Humphrey Field Analyzer (SITA FAST 24-2). Mean detection thresholds for each eye were calculated, along with quadrants and central measures. Retinal layers were manually segmented in the macular region.

**Results:**

Mean detection thresholds were significantly lower than normative values for PwA (−3.10 ± 1.67 dB, *P* << 0.0001) and PwIIN (−1.70 ± 1.54 dB, *P* < 0.0001). Mean detection thresholds were significantly lower in PwA compared to PwIIN (*P* < 0.0001) and significantly worse for left compared to right eyes in PwA (*P* = 0.0002) but not in PwIIN (*P* = 0.37). In PwA, the superior nasal VF was significantly worse than other quadrants (*P* < 0.05). PwIIN appeared to show a mild relative arcuate scotoma. In PwA, central detection thresholds were correlated with foveal changes in the inner and outer retina. VF was strongly correlated to BCVA in both groups.

**Conclusions:**

Clear peripheral and central VF deficits exist in PwA and PwIIN, and static VF results need to be interpreted with caution clinically. Since PwA exhibit considerably lower detection thresholds compared to PwIIN, VF defects are unlikely to be due to nystagmus in PwA. In addition to horizontal VF asymmetry, PwA exhibit both vertical and interocular asymmetries, which needs further exploration.

Albinism is a group of inherited disorders in melanin biosynthesis associated with a range of visual system abnormalities, including high refractive errors,^1^ iris transillumination,^2^ foveal hypoplasia,^3^ thinning of the circumpapillary retinal nerve fiber layer (cpRNFL),^4^ optic nerve head abnormalities,^4^ chiasmal misrouting,^5^^–^^7^ and nystagmus.^8^ The development of high-resolution three-dimensional imaging technologies of the eye, including optical coherence tomography (OCT)^9^ and adaptive optics,^10^ has led to foveal deficits being well characterized in people with albinism (PwA). For example, a recent study reports that oculocutaneous albinism is associated with all four grades of foveal hypoplasia, whereas ocular albinism and FHONDA (albinism-related conditions with normal pigmentation) are associated with more severe foveal hypoplasia.^3^

Peripheral vision in PwA is less well understood. There is a paucity of literature of visual field (VF) testing in PwA, especially in the use of static perimetry. One problem is that the effect of nystagmus is unclear since peripheral vision is particularly sensitive to retinal motion.

Nasotemporal asymmetries can be seen throughout the visual system in PwA. In the retina, the line of decussation is shifted toward the temporal retina instead of falling along the vertical meridian through the fovea.^11^ Also, marked nasotemporal asymmetry in the ganglion cell layer (GCL) distribution around the fovea can be observed using OCT measurements due to thicker GCL on the nasal compared to the temporal aspect.^12^^–^^14^ Misrouting of retinal ganglion cell axons through the optic chiasm leads to an abnormal projection of the ipsilateral visual field from the temporal retina being superimposed upon the normal representation of the contralateral visual field projecting from the nasal hemiretina.^15^^,^^16^

Previous studies on albinism, including studies of VF deficits, often use people with idiopathic infantile nystagmus (PwIIN) as a comparator group,^17^^,^^18^ since nystagmus waveforms are similar^8^ yet retinal deficits appear to be much less severe. While this is true in respect to central vision, with PwIIN showing either mild (grade 1) or no foveal hypoplasia, the effect of idiopathic infantile nystagmus (IN) on peripheral vision is unclear. Structural^19^ and functional^20^^–^^22^ deficits in the retina exist outside the fovea in human and animal models of idiopathic IN.

Three previous studies have assessed visual fields in either PwA and/or PwIIN, mostly using kinetic rather than static perimetry and using small sample sizes. St John and Timney^18^ assessed the VFs of 13 PwA and 15 controls using a tangent screen (i.e., kinetic perimetry), also testing contrast sensitivity using sine wave gratings. They found 9 of 13 PwA had contracted VFs and that contrast sensitivity was reduced compared to controls. The worst affected PwA had poorer nasal compared to temporal VFs.

Abadi and Pascal^17^ assessed the VFs of 11 PwA, 6 PwIIN, and 6 controls using Goldman VFs, another type of kinetic perimetry. They observed no constriction of VFs in either PwA or PwIIN and no temporal-nasal asymmetries. They also reported worse contrast sensitivity for PwA compared to either PwIIN or controls.

Hoffmann et al.^23^ assessed static perimetry in 15 PwA and 6 controls using the Octopus 101 (Haag-Streit, Köniz, Switzerland) instrument. They reported no VF size differences and no temporal-nasal asymmetries. They reported reduced contrast sensitivity around the blind spot in PwA. To date, no study has compared PwA with PwIIN using static perimetry.

Given the clear visual pathway nasotemporal asymmetries in PwA and that previous literature is ambiguous in relation to nasotemporal VF asymmetries, we have used a larger sample size than previous studies including 62 PwA and 36 PwIIN to investigate horizontal VF asymmetries. In addition, we have also compared the relative size of vertical VF asymmetries and left eye – right eye VF differences. As we observed marked interocular VF asymmetries in PwA, we also compared interocular differences in eye dominance.

Since significant changes in retinal architecture are evident on OCT in PwA, we have also compared VF and OCT measures, namely: (1) central VF measurements and OCT measures of the foveal region, (2) central and peripheral VF measurements to cpRNFL thinning, and (3) nasotemporal VF asymmetry to nasotemporal asymmetry of the ganglion cell complex (GCC) in PwA. We have used GCC thickness measures rather than GCL thickness because they can be segmented more accurately on single B-scans. VF measurements have also been compared to best-corrected visual acuity (BCVA) measurements in both groups.

## Methods

### Participants

Sixty-one PwA (44 male, 17 female, mean age = 32.4 years, range 12–66 years with three participants <16 years) and 32 PwIIN (24 male, 8 female, mean age = 33.9 years, range 13–66 years with two participants <16 years) were included in the study. All participants were recruited from adult and pediatric ophthalmology clinics at the Leicester Royal Infirmary, United Kingdom. Informed consent was obtained before examination. For participants <16 years of age (*n* = 4), parental/guardian consent was obtained. The study adhered to the tenets of Declaration of Helsinki and was approved by the local ethics committee.

All patients had a thorough eye examination, including BCVA (where the preferred head posture was adopted), ocular motility, stereopsis, slit-lamp biomicroscopy (to assess fundus hypopigmentation and iris transillumination), OCT imaging to determine foveal hypoplasia (see later description), and visual evoked potentials to detect chiasmal misrouting (five-channel, pattern onset/offset). Eye movements were recorded under binocular conditions on all participants at primary position (500 Hz; EyeLink II pupil tracker, SR Research Ltd., Ottawa, Canada) and calibrated offline using procedures previously described.^24^ Intensity of the nystagmus was calculated as amplitude × intensity of nystagmus over an average of 1-minute recordings of the data.

Albinism was confirmed using criteria developed by Kruijt et al.,^25^ with positive confirmation through the presence of either three major criteria or two major and two minor criteria, where major criteria were (1) grade 2 or more foveal hypoplasia, (2) misrouting confirmed using visual evoked potentials (VEP), and (3) ocular hypopigmentation (either iris translucency or fundus hypopigmentation grade 2 or more). Minor criteria were (1) nystagmus, (2) hypopigmentation of skin and hair, (3) grade 1 fundus hypopigmentation, and (4) foveal hypoplasia grade 1. Diagnosis of idiopathic IN was confirmed by the presence of nystagmus with no VEP crossing abnormality, no iris transillumination, and only grade 1 or no fovea hypoplasia on OCT.

### Visual Field Assessments Using Static Perimetry

The VF was assessed using the Humphrey Field Analyser (HFA; Carl Zeiss Meditec AG, Cambridge, UK) using a SITA (Swedish Interactive Threshold Algorithm) Fast 24-2 method (size = Goldmann III). Detection thresholds were obtained for 54 locations up to a 24° radius around the fixation point. Each value represents the deviation, in decibels (dB), from normative values obtained by the manufacturer for the device, which is corrected for age. The order of testing was right eye followed by left, following the manufacturer’s instructions. All participants wore refractive correction, where required, during VF testing.

**Figure 1.**
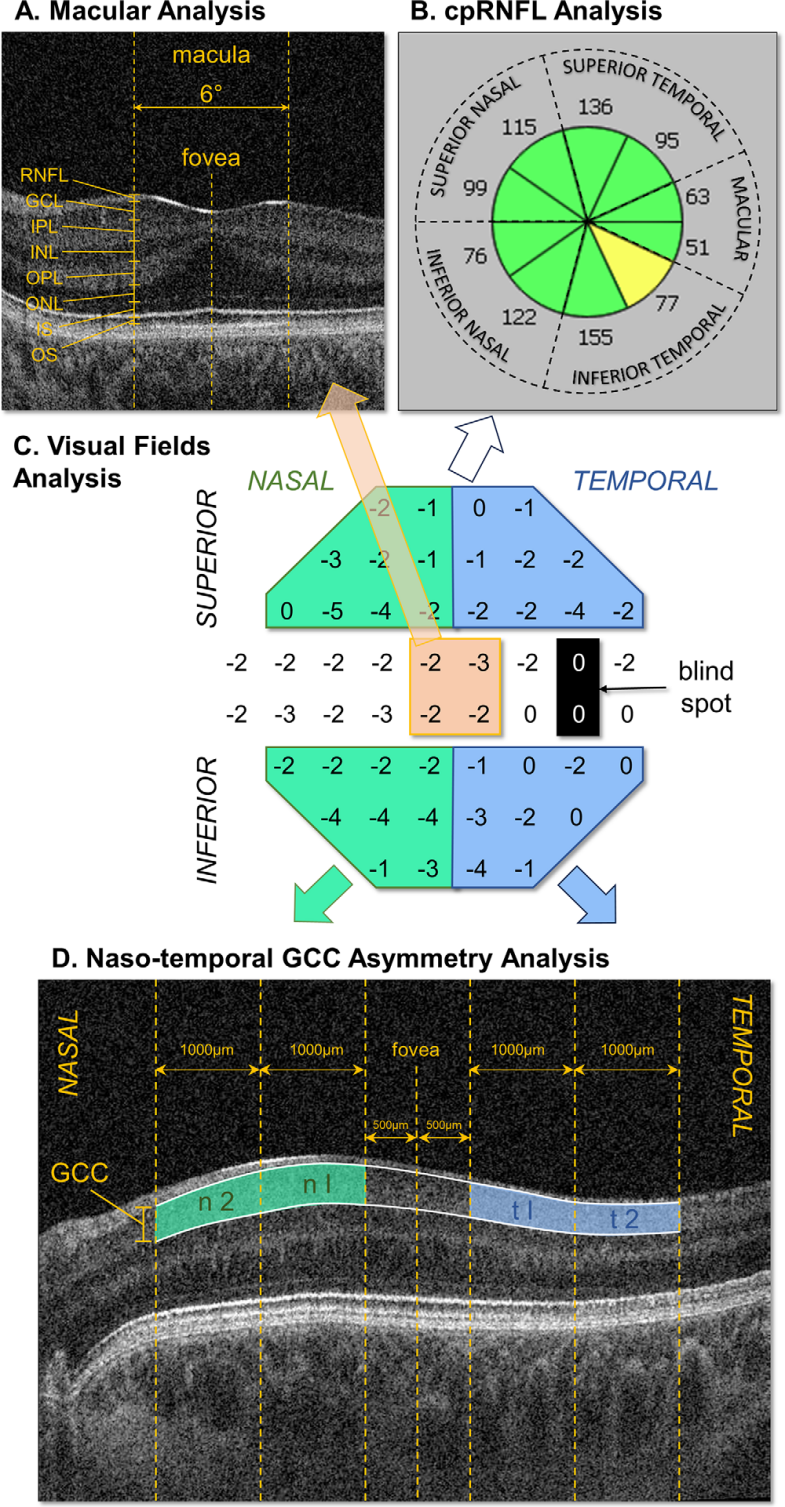
Summary of the VF and OCT analysis approach used. The central panel shown in (**C**) shows the points averaged on the VF maps for the quadrant and hemifield analysis, with superior and inferior nasal VFs in *green*, temporal VFs in *blue*, and central VFs in *orange*. OCT analysis was performed on PwA where (**A**) shows the region analyzed on OCT foveal B-scan images comparing retinal layer thicknesses: (1) at the center of the fovea and (2) in the 6° region corresponding to the central VF (in *orange*). (**B**) Location of the radial segments used for the cpRNFL OCT analysis with thickness averaged over an anulus from 2.4 to 3.2 mm in diameter (GDx Nerve Fiber Analyzer protocol; Carl Zeiss Meditec). The numbers shown are examples of mean RNFL thickness (in µm) for one individual where *green* indicates normal and *amber* RNFL thinning outside of the 95% confidence interval. (**D**) Areas analyzed to compare the nasal-temporal asymmetry of the GCC distribution around the fovea. INL, inner nuclear layer; IPL, inner plexiform layer; IS, inner segments; OPL, outer plexiform layer; OS, outer segments.

Mean detection thresholds were calculated for each eye across all 52 positions (excluding the two testing points at the blind spot, Fig. 1C). Superior and inferior, as well as nasal and temporal, VF quadrants were also estimated from the mean values of the positions as indicated in Figure 1C, avoiding the two rows across the horizontal midline to minimize artifacts caused by the blind spot. Central detection thresholds were also estimated from the four central VF positions shown in orange in Figure 1C.

### Optical Coherence Tomography Acquisition and Analysis in PwA

Foveal scans were acquired from both eyes of PwA, where possible, using spectral domain OCT (SOCT Copernicus HR, Optopol Technology S.A., Zawiercie, Poland; 3-µm axial resolution, 52,000-Hz A-scan/s, 7-mm × 7-mm scan, 743 A-scans, 75 B-scans). Foveal B-scans of sufficient quality to allow segmentation were obtained from 99 eyes of 54 PwA and 54 eyes of 27 PwIIN.

For foveal analysis, retinal layers were manually segmented on B-scans through the foveal center using an ImageJ macro (available at http://rsbweb.nih.gov/ij/; accessed November 5, 2021) to determine retinal layer thickness, as indicated in Figure 1A. Measurements at the foveal center and in the macular region (corresponding ±6° around the foveal center, equivalent to ±892 µm on the OCT B-scan) were compared to central detection thresholds.

cpRNFL segmentation was also carried out, after realignment of B-scan images using the method described in Mohammad et al.^4^ (i.e., annulus: 2.4 to 3.2 mm diameter, 10 radial segments using the GDx Nerve Fiber Analyzer protocol, Carl Zeiss Meditec, Fig. 1B). Five radial segments, including the (1) superior nasal, (2) inferior nasal, (3) superior temporal, (4) inferior temporal, and (5) the macular segment, were analyzed and correlated with VF quadrants and central detection thresholds.

To compare the nasal-temporal asymmetry of the GCC to VF measures in PwA, we based our approach on that developed by Brucher et al. and others^12^^–^^14^ with some modifications. These include (1) using the GCC (ganglion cell layer + inner plexiform layer) thickness, which can be more accurately determined than GCL thickness on single B-scans, and (2) setting the outer limit at 5 mm rather than 6 mm to reduce clipping caused by the B-scan not being centrally located because of nystagmus. The GCCT-I-Quotient and GCCT-II-Quotient are defined as mean GCC thickness in t I/n I and t II/n II, respectively, as shown in Figure 1D. GCCT-II-Quotient could not be determined on two participants because of clipping.

### Eye Dominance

Eye dominance was determined in PwA by requesting participants to roll up a sheet of paper and, with two hands, bring it up to one eye to look through.

### Statistical Analysis

One-sample two-tailed *t*-tests were used to compare mean deviations to zero (i.e., the normative age-matched mean detection thresholds), and *z-*scores were also plotted to indicate the level of deviation away from normal. Unpaired and paired *t*-tests were used to compare mean deviations for each eye between the groups and left/right eyes, respectively. Linear mixed models were used to compare VF asymmetries, quadrant analysis, and eye dominance, including eye as a factor, to investigate potential interactions between eye and VF. VF asymmetries and quadrant comparisons were also performed after excluding outliers using the Tukey method. Comparisons of quadrants in each eye were made using Friedman's test with Dunn's correction for multiple comparisons to avoid potential artifacts caused by outliers. OCT and BCVA measures were correlated with VF measures using Pearson correlation to determine the direction of associations, using linear mixed models to determine *P* values.

## Results

### Detection Thresholds for Both Eyes in PwA and PwIIN

**Figure 2.**
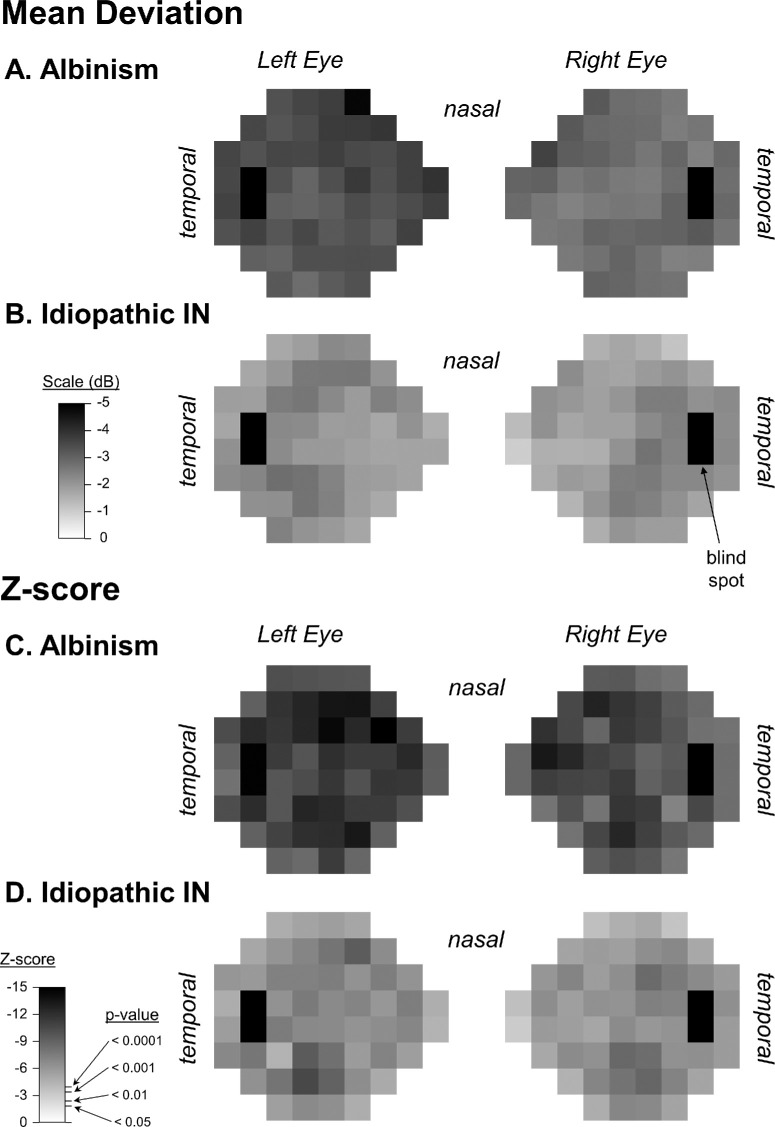
Mean deviation detection thresholds for all 54 locations in left and right eyes of (**A**) PwA and (**B**) PwIIN. Statistical comparisons to normative values are shown in (**C**) and (**D**), respectively, where *z*-scores for one-sample *t*-tests are shown. The *P* value equivalents are also indicated. Each square represents 6° of the visual field, with darker squares indicating poorer detection thresholds. The blind spot is represented by the *black squares* toward the temporal aspect of visual fields.

Detection thresholds for all 52 test locations in each eye are shown in Figures 2A and 2B for PwA and PwIIN, respectively, with the statistical differences compared to mean normative values represented in Figures 2C and 2D, respectively.

For PwA, all 52 locations in both eyes were significantly lower than normal by at least 2 dB. Mean ± SD detection thresholds for right and left eyes combined were –3.10 ± 1.67 dB for PwA (*t*(61) = 14.7, *P* << 0.0001). In PwIIN, 96% of test points were significantly lower than normal. Mean detection thresholds were –1.70 ± 1.54 dB (*t*(96) = 6.62, *P* < 0.0001). Mean detection thresholds were significantly lower in PwA compared to PwIIN (*t*(96) = 4.14, *P* < 0.0001).

Using the statistical comparisons provided by the Humphrey machine, for PwA, 83.9% of left eye VFs were below the 5% confidence interval (CI) and 66.1% of right eye VFs (Table 1). For PwIIN, 44.4% of left eye VFs for PwA were below the 5% CI and 41.7% of right eye VFs.

### Visual Field Asymmetries

Mean deviations were more negative for nasal compared to temporal hemifields in PwA showing borderline significant differences (Table 2). The mean temporal to nasal difference was 0.275 dB in right eyes (95% CI, 0.058–0.492 dB, *P* = 0.020) and 0.277 dB in left eyes (95% CI, 0.014–0.561 dB, *P* = 0.061). Nasal temporal VF differences did not reach statistical significance in PwIIN (*P* = 0.113 and 0.46 for right and left eyes, respectively). The left eyes of PwA also demonstrated a significant vertical asymmetry due to more negative mean deviations in superior fields (0.460 dB, 95% CI, 0.093–0.826 dB, *P* = 0.020, right eye mean deviation 0.105, *P* = 0.547).

**Figure 3.**
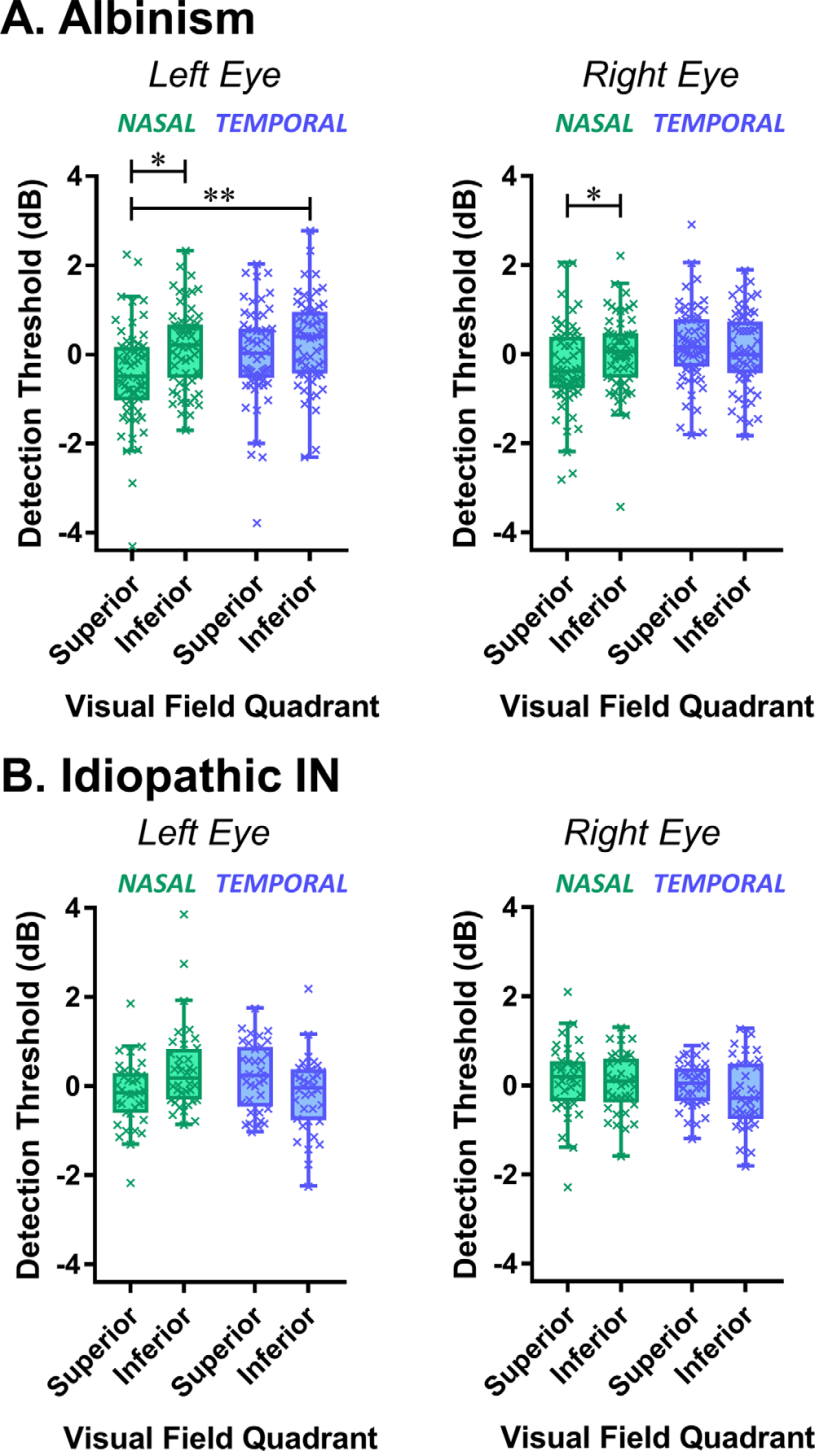
Box-and-whisker plots of the within-subject differences in visual field quadrants (i.e., where mean deviations for each quadrant are expressed relative to the mean of all four quadrants). The box and whiskers indicate median, quartiles, and the range excluding outliers determined using the Tukey method. Quadrant comparisons for each eye in each group were made using Friedman's tests and Dunn's test for multiple comparisons. IN, infantile nystagmus.

Within-subject differences between superior and inferior, as well as temporal and nasal, quadrants are represented in Figure 3, with statistical comparisons comparing VF asymmetries and quadrant differences shown in Table 2. The analysis was also repeated after excluding outliers using the Tukey method (Supplementary Table S1).

For PwA, horizontal and vertical VF asymmetries were mainly due to reduced sensitivity in the superior nasal VF compared to other quadrants. The pattern was more obvious in the left eye compared to the right eye (Fig. 3A), but there were no significant interactions between eye and quadrant (Table 2).

There was a significant interaction between horizontal and vertical VF asymmetry in PwIIN, although not after the exclusion of outliers (Supplementary Table S1). This was caused by a greater VF deficit in temporal compared to nasal inferior VF. This pattern was reversed in the superior VF of left eyes with no difference between nasal and temporal VF in right eyes (Fig. 3B). This was a subtle effect with quadrant differences being near threshold for significance (*P* = 0.079, Table 2). The mean deviation plot show in Figure 1B suggests a mild relative arcuate scotoma in PwIIN visible in the temporal inferior VFs of both eyes but extending across temporal and nasal superior VFs of the left eye.

### Central Detection Thresholds

Central detection thresholds (indicated on Fig. 1 in orange) were significantly lower than normative values in both PwA (*t*(61) = 12.1, *P* << 0.0001) and PwIIN (*t*(96) = 6.95, *P* < 0.0001). Central detection thresholds were significantly lower in PwA (−2.73 ± 1.77 dB) compared to PwIIN (−1.80 ± 1.56 dB, *t*(96) = 2.61, *P* = 0.010).

### Comparison of Left and Right Eye Visual Fields

Mean detection thresholds were significantly worse for left compared to right eyes for PwA (mean difference: 0.841 dB; 95% CI, 0.418–1.264 dB; *t*(61) = 3.89; *P* = 0.0002) but not for PwIIN (mean difference: 0.167 dB; 95% CI, –0.192 to 0.526 dB; *t*(36) = 0.91, *P* = 0.37; Table 1^2^). Similarly, mean detection thresholds at the macular were significantly worse for left compared to right eyes for PwA (*t*(61) = 2.56, *P* = 0.013) but not for PwIIN (*t*(35) = 0.78, *P* = 0.44). The left–right eye differences in PwA were consistent across all regions of the VF but were more obvious in superior VF. Upper nasal VFs were also significantly lower in PwIIN in the left eye VFs.

**Table 1.** Mean Deviations With 95% CI of Left (L) and Right (R) Eyes of People With Albinism and People With Idiopathic Infantile Nystagmus for Full VFs Along With Central VFs, Quadrants, and BCVA

	Left Eyes			Right Eyes			L Compared to R	
		95% CI			95% CI			
Characteristic	Mean Deviation (dB)	Lower	Upper	Mean Deviation (dB)	Lower	Upper	*T*	*P*
**Albinism**								
Full VF	−3.52	−7.44	0.40	−2.68	−6.06	0.69	3.89	0.000
Percentage of mean deviations identified as abnormal by the Humphrey VF machine								
<5% CI	83.9%			66.1%			—	—
<1% CI	58.1%			41.9%			—	—
<0.5% CI	12.9%			3.2%			—	—
Central	−3.08	−7.47	1.30	−2.38	−6.12	1.36	**2.56**	**0.013**
Superior nasal	−4.00	−8.21	0.22	−3.03	−6.67	0.61	**4.04**	**0.000**
Inferior nasal	−3.37	−7.54	0.81	−2.70	−6.54	1.13	**2.44**	**0.018**
Superior temporal	−3.55	−7.90	0.80	−2.53	−6.50	1.43	**4.00**	**0.000**
Inferior temporal	−3.26	−7.77	1.25	−2.65	−6.68	1.39	**2.34**	**0.023**
BCVA	0.53	0.10	0.96	0.52	0.14	0.90	0.70	0.485
**Idiopathic infantile nystagmus**								
Full VF	−1.78	−4.97	1.40	−1.62	−4.85	1.62	0.91	0.370
Percentage of mean deviations identified as abnormal by the Humphrey VF machine								
<5% CI	44.4%			41.7%			—	—
<1% CI	19.4%			19.4%			—	—
<0.5% CI	2.8%			0.0%			—	—
Foveal	−1.67	−4.89	1.54	−1.93	−5.90	2.04	0.78	0.439
Superior nasal	−2.15	−6.18	1.88	−1.51	−5.44	2.42	**2.77**	**0.009**
Inferior nasal	−1.60	−4.75	1.55	−1.59	−4.67	1.49	0.04	0.967
Superior temporal	−1.76	−5.55	2.04	−1.64	−5.14	1.86	0.50	0.617
Inferior temporal	−2.16	−5.65	1.33	−1.84	−5.37	1.69	1.23	0.227
BCVA	0.30	−0.06	0.65	0.27	0.03	0.50	1.36	0.182

The percentage of individuals highlighted as demonstrating abnormal VF by the Humphrey machine is also shown. Statistical comparisons of left and right eyes using paired *t*-tests are shown on the right with significant difference highlighted in bold text.

**Table 2.** Results of Linear Mixed Models, Including Left and Right Eye Data, Comparing Visual Field Horizontal and Vertical Asymmetries and the Four Quadrants With Respect to Each Other

	Horizontal (Nasal – Temporal)		Vertical (Superior – Inferior)		Horizontal × Vertical Interaction	
Characteristic	*F*	*P*	*F*	*P*	*F*	*P*
Horizontal and vertical asymmetry analysis						
Albinism	**5.213**	**0.023**	**5.444**	**0.020**	2.585	0.109
Idiopathic infantile nystagmus	1.382	0.241	0.080	0.778	**5.399**	**0.021**
	**Quadrant**		**Post Hoc Comparison (*P* Values)**			
	***F***	***P***	**SN – IN**	**SN – ST**	**SN – IT**	

Quadrant analysis						
Albinism	**4.414**	**0.005**	**0.033**	**0.037**	**0.007**	
Idiopathic infantile nystagmus	2.287	0.079	NS	NS	NS	

Only significant quadrants differences are shown. Significant differences are indicated in bold. I, inferior; N, nasal; NS, not significant; S, superior, T, temporal.

### Comparison of Dominant and Nondominant Eye Visual Fields in PwA

Eye dominance was explored in PwA as the potential cause of interocular differences in VF detection thresholds. Thirty-six PwA were determined as right eye dominant and 26 as left eye dominant. Mean detection thresholds were significantly worse for nondominant compared to dominant eyes in PwA (mean difference: 0.874 dB; 95% CI, 0.455–1.292 dB, *P* = 0.020; *t*(61) = 4.09; *P* = 0.0001). There was a significant interaction between eye and eye dominance (*F*(57) = 11.29, *P* = 0.014). This was due to detection thresholds being significantly worse in the left eyes of PwA who were right eye dominant (59.3%: left: –3.92 ± 1.97 dB; right: –2.46 ± 1.55 logMAR; *t*(35) = 1.69, *P* < 0.0001) compared to those who were left eye dominant. These participants showed no significant interocular difference in detection thresholds (40.7%: left: –3.00 ± 1.93 logMAR; right: –2.83 ± 1.83 logMAR; *t*(23) = 1.07, *P* = 0.29).

### Relationship Between Detection Thresholds and OCT Measures in PwA

Central detection thresholds were negatively correlated to retinal nerve fiber layer (RNFL) and inner nuclear layer thickness and positively correlated to outer nuclear layer (ONL) and outer segment (OS) thickness at the center of the fovea (*r* values ranging from –0.28 to –0.32, Table 3). Central detection thresholds were also correlated negatively to RNFL and positively to ONL thickness across the macular region. There were no significant correlations between cpRNFL thickness and VF for the whole eye, central detection thresholds, or any quadrants (Supplementary Table S2). There were also no significant correlations between either GCCT-I-Quotient or GCCT-II-Quotient and foveal VF detection thresholds or the nasotemporal asymmetry in VF detection thresholds (Supplementary Table S3).

**Table 3.** Correlations Between Central Detection Thresholds and Retinal Layer Thicknesses at the Center of the Fovea and Macula for PwA (±6° Either Side of the Fovea, i.e., the Area Equivalent to the VF Test Locations)

	Fovea		Macula	
Characteristic	*r*	*P*	*r*	*P*
Inner retina				
RNFL	**0.31**	**0.002**	**0.33**	**0.001**
GCL	0.01	0.899	0.11	0.276
IPL	0.08	0.451	0.04	0.691
INL	**0.28**	**0.005**	0.09	0.403
Outer retina				
OPL	0.03	0.797	0.02	0.860
ONL	**0.32**	**0.001**	**0.30**	**0.003**
IS	0.16	0.109	0.15	0.144
OS	**0.28**	**0.005**	0.11	0.273

The *P* values are generated from linear mixed models to account for left and right eyes. INL, inner nuclear layer; IPL, inner plexiform layer; IS, inner segments; OPL, outer plexiform layer; OS, outer segments.

### Relationship Between Detection Thresholds and BCVA

Both central detection thresholds and whole-eye detection thresholds were strongly correlated with BCVA for both PwA (central: *r* = –0.49, slope = –0.048 (95% CI, –0.033 to –0.064), *P* << 0.0001; whole eye: *r* = –0.47, slope = –0.050 [95% CI, –0.033 to –0.068], *P* < 0.0001) and PwIIN (central: *r* = –0.45, slope = – 0.038 [95% CI, –0.020 to –0.057], *P* = 0.0001; whole eye: *r* = –0.42, slope = –0.041 [95% CI, –0.020 to –0.062], *P* = 0.0001). Despite the clear differences in left and right eye VFs for PwA, there were no significant interocular differences in BCVA (Table 1, *t*(61) = 0.22, *P* = 0.82).

### Nystagmus

Intensity of nystagmus under binocular conditions was similar between the two groups (mean ± SD: 16.13 ± 12.19°/s in PwA; 17.70 ± 10.82°/s in PwIIN, *P* = 0.52).

## Discussion

### Conclusion

For both PwA and PwIIN, detection thresholds were significantly lower than normative mean values across all regions of the VF tested. However, detection thresholds were significantly lower in PwA compared to PwIIN, indicating that VF defects in PwA are unlikely to be caused by nystagmus. Small yet significantly reduced VF detection thresholds were observed in nasal VF of PwA compared to temporal VF. However, these differences were much smaller than interocular differences, either expressed as left–right eye differences or dominant–nondominant eye differences. Left eye detection thresholds were significantly worse than for right eyes in PwA. This pattern was more obvious in the superior VFs and in PwA who had dominant right eyes. This interocular asymmetry was not observed in PwIIN or in BCVA measures in PwA.

PwA showed a specific VF deficit in the superior nasal VF compared to other VF quadrants, whereas PwIIN demonstrated what appeared to be a mild relative arcuate VF deficit (Fig. 2B). Although central detection thresholds were correlated to foveal OCT measurements, the correlation to BCVA was much stronger, and there were no significant correlations between VF and cpRNFL measures.

### Detection Thresholds for PwA and PwIIN

Of the three previous studies assessing VF in PwA or PwIIN, two studies used kinetic perimetry,^17^^,^^18^ reporting conflicting results concerning whether VFs are contracted in PwA.^17^ Only one previous study used static perimetry (Octopus 101 instrument) on a small number of PwA (*n* = 15) compared to six controls reporting no selective visual field defects corresponding to the abnormally projecting temporal retina.^23^ In this study, we used a standard protocol (SITA Fast 24-2 method) on the Humphrey Field Analyser, one of the most widely used VF clinical devices. We observed very clear VF deficits in both PwA and PwIIN compared to mean age-adjusted normative values, using one-sample *t*-tests or using the statistical comparisons provided by the Humphrey machine. These findings indicate clear widespread deficits across the peripheral VF up to 24° around the fovea in both groups but especially in PwA.

Mean deviations were 82% greater in magnitude for PwA (−3.10 ± 1.67 dB) compared to PwIIN (−1.70 ± 1.54 dB, *P* < 0.0001). Idiopathic IN cannot really be considered a control group for albinism, since anatomic and functional retinal abnormalities are now known to exist in PwIIN.^19^^,^^20^^,^^22^ The lower detection thresholds in PwA compared to PwIIN, however, indicate that VF deficits in PwA are not primarily caused by the presence of nystagmus, which is similar in the two groups.^8^

As a psychophysical test, VF testing reflects visual function from the retina through to the cortex. Retinal deficits are reported in both PwA^9^ and PwIIN.^19^^,^^20^^,^^22^ In addition, deficits exist in PwA in the optic nerve head,^4^ along the visual pathway to the brain (optic nerves, chiasm, and tracts),^5^^–^^7^ and in the structure^5^ and connectivity of the visual cortex.^16^^,^^26^^–^^28^ The relative contribution of these deficits is unclear at this stage. The absence of any correlation between cpRNFL thickness and VF measurements may indicate the importance of extraretinal factors. In addition, central detection thresholds were strongly correlated to BCVA than foveal OCT measures, a measure that also captures changes along the visual pathway.

### Nasotemporal Visual Field Asymmetries

The use of a larger sample in this study has addressed some of the ambiguities of previous literature. Small yet significant reduced VF detection thresholds were observed in nasal VF of PwA compared to temporal VF, with the difference reaching significance in right eyes (*P* = 0.020) but borderline significance in left eyes (*P* = 0.061). These differences may have been missed in previous studies using smaller sample sizes. We did not observe, however, a significant correlation between the naso-temporal asymmetry of the GCC around the fovea and VF naso-temporal asymmetry (Supplementary Table S3).

An obvious cause of these nasotemporal VF differences is that the abnormal projection of the ipsilateral visual field from the temporal retina is less sensitive that the normal contralateral visual field projecting from the nasal hemiretina.^15^^,^^16^ It is surprising that these nasotemporal VF differences are not greater given the structural differences that can been seen in the retina,^12^^–^^14^ optic nerve head,^4^ chiasm,^5^^–^^7^ and cortex.^5^^,^^15^^,^^16^ This may be an indication of the ability of the developing visual system to adapt to significant structural changes.

### Quadrant Visual Field Deficits

Previous VF studies disagree in relation to whether nasal-temporal VF asymmetry exists in PwA.^17^^,^^18^^,^^23^ For PwA, the most obvious pattern was a specific superior nasal VF deficit compared to other VF quadrants. This is equivalent to an inferior temporal deficit in the retina and the areas of the cortex it projects to. In the normal retina, ipsilateral retinal ganglion cell axons originating in the temporal retina are thought to emerge earlier than crossed axons in the nasal retina.^29^ Delayed neurogenesis in PwA may lead to not only a reduction in the proportion of uncrossed axons^30^ but also possibly lower populations of retinal ganglion cells (RGCs) in the temporal retina, or reduced connectivity cortically, compared to normal. This is supported by the disproportionately thinner cpRNFL in PwA on the temporal aspect of the optic nerve head, although it should also be borne in mind that this region of the cpRNFL also projects from the macula.^9^ These findings also highlight the importance of considering vertical and well as horizontal retinal asymmetries.

Horizontal and vertical asymmetries were weaker for PwIIN, although a subtle arcuate VF deficit was most obviously seen in the left eye. The darkened region in the left eye of Figure 2B appears to follow the trajectory of the superior and inferior arcades. Interestingly, changes in retinal branching patterns have been described in both PwA and PwIIN.^31^

### Visual Field Differences Between Eyes

One of the clearest patterns observed was the lower detection thresholds in the left eyes of PwA compared to right eyes (mean difference 0.841 dB) and worse in nondominant eyes compared to dominant eyes (0.874 dB) (Table 1). This was three times larger in magnitude compared to nasotemporal differences in VF. We followed the manufacturer's instructions in testing the right eye first, followed by the left. Although this could introduce a confounder, there were no interocular VF differences in PwIIN (*P* = 0.37), suggesting that the order of testing was not important.

In a previous study, we have observed that cpRNFL was significantly thicker in right compared to left eyes of PwA (*P* = 0.001).^9^ Differences in cpRNFL were not related to eye dominance (*P* = 0.32), in contrast to our findings. Cross-sectional areas of the left and right optic tracts in PwA are similar, suggesting that inputs to each cortical hemisphere are approximately equal.^32^

A consequence of chiasmal misrouting is the greater bias toward monocular inputs feeding into each visual hemisphere compared to the normal situation where approximately equal inputs come from each eye.^16^ In the normal population, left–right hemispheric cortical asymmetries exist for visual attention, a feature clearly seen in visuospatial hemineglect of the left hemifield following lesions to the right parietal lobe.^33^ Right-sided visuospatial neglect is rarer following left parietal lobe damage since the right parietal lobe processes information from right and left hemifields. Inhibitory transcranial magnetic stimulation of the right parietal lobe also leads to selective inattention of the left hemifield.^34^ Possibly the interocular VF asymmetries apparent in PwA are caused by predominantly monocular inputs accentuating hemispheric differences, although the reduced sensitivity of left eye VF detection thresholds apparently does not match the more important role of the right parietal lobe in visuospatial attention. These data highlight the importance that interocular developmental differences should be investigated in future studies in PwA. Interocular differences, although less obvious, were seen in PwIIN but only for the superior nasal quadrant (Table 1).

Our study mainly included adults, with only five participants under the age of 16 years. Recently, OCT measurements have been used to estimate the trajectory of retinal development in PwA showing that the outer retina in particular shows delayed development compared to controls, continuing until at least 6 years of age.^35^ It would be interesting to investigate the development of VF in early childhood and whether the patterns observed in adulthood, such as horizontal and vertical asymmetries in detection thresholds, are more obvious early in life. However, it would be a challenging to detect these small differences in young children. The intensity of nystagmus under binocular conditions was similar between the two groups (*P* = 0.52). However, visual fields were measured under monocular conditions, and the possibility of different proportions of fusion maldevelopment nystagmus syndrome (or latent nystagmus) between PwA and IIN groups cannot be ruled out as a cause of different nystagmus characteristics under monocular conditions.

### Conclusions

In conclusion, static VF mean detection thresholds are significantly lower than normal in PwA and PwIIN. Hence, VF results need to be interpreted with some caution in the clinic. Since lower detection thresholds exist in PwA compared to PwIIN, the VF defects in PwA are unlikely to be due to nystagmus as the nystagmus is similar in the two groups.^8^ The superior nasal VF is the worst affected quadrant in PwA, and PwIIN also appear to demonstrate a mild arcuate scotoma. Overall small yet significantly reduced VF sensitivities were observed in nasal compared to temporal VF of PwA, settling previous ambiguities. These findings have clinical importance in the interpretation of visual fields in these conditions but also reveal two areas that have been largely ignored, that of a clear interocular asymmetry in the peripheral fields of PwA and a vertical component to retinal asymmetry in addition to the well-known horizontal asymmetry.

## Supplementary Material

Supplement 1

Supplement 2

Supplement 3
