# Supplementary material for: Visual Field Deficits in Albinism in Comparison to Idiopathic Infantile Nystagmus
Source: Invest Ophthalmol Vis Sci. 2024 Feb 6;65(2):13. doi: 10.1167/iovs.65.2.13 (PMC10854418; doi:10.1167/iovs.65.2.13)
Supplement: Supplement 1 [file iovs-65-2-13_s001.pdf]

***Supplementary Table 1:*** *The results of linear mixed models, after excluding outliers using the Tukey method, comparing (A) visual field horizontal and vertical asymmetries and (B) the four quadrants with respect to each other (only significant quadrants differences are shown). IN = infantile nystagmus. S=superior, I=inferior, N=nasal and T= temporal visual fields. NS = not significant. Significant differences are indicated in bold.*

## A. Horizontal and Vertical Asymmetry Analysis

| | HORIZONTAL (nasal - temporal) | | VERTICAL (superior - inferior) | | Horiz. * Vert. interaction | |
|---|---|---|---|---|---|---|
| | *F* | *P* | *F* | *P* | *F* | *P* |
| **Albinism** | **7.796** | **0.005** | **4.254** | **0.040** | **4.119** | **0.043** |
| **Idiopathic-IN** | 2.126 | 0.146 | 0.717 | 0.398 | 3.856 | 0.051 |

## B. Quadrant Analysis

| | Quadrant | | **Post-hoc comparison** (*P*-values) | | |
|---|---|---|---|---|---|
| | *F* | *P* | **SN - IN** | **SN - ST** | **SN - IT** |
| **Albinism** | **5.415** | **0.001** | **0.004** | **0.024** | **0.004** |
| **Idiopathic-IN** | 2.253 | 0.083 | NS | NS | NS |
