# Supplementary material for: Visual Field Deficits in Albinism in Comparison to Idiopathic Infantile Nystagmus
Source: Invest Ophthalmol Vis Sci. 2024 Feb 6;65(2):13. doi: 10.1167/iovs.65.2.13 (PMC10854418; doi:10.1167/iovs.65.2.13)
Supplement: Supplement 2 [file iovs-65-2-13_s002.pdf]

Supplementary Table 2: *The table shows that there were no significant correlations between circumpapillary retinal nerve fiber (cpRNFL) layer thickness measurements and visual field measurements in PwA. The segments of the cpRNFL compared are shown in* ***Figure 1****. Data for Left and right eyes are shown in* ***(A)*** *and* ***(B)****, respectively.*

**A. Left Eye**		**CIRCUMPAPILLARY RETINAL NERVE FIBER LAYER**					
**VISUAL FIELD**		**Full**	**Superior nasal**	**Inferior nasal**	**Superior temporal**	**Inferior temporal**	**Macular**
**Full**	*r*	0.257	0.173	0.298	0.165	0.026	0.076
	P	0.135	0.320	0.082	0.344	0.884	0.665
**Superior nasal**	*r*	0.285	0.135	0.280	0.172	0.135	0.084
	P	0.097	0.439	0.103	0.323	0.440	0.632
**Inferior nasal**	*r*	0.190	0.169	0.243	0.122	-0.020	0.031
	P	0.274	0.331	0.159	0.485	0.910	0.860
**Superior temporal**	*r*	0.308	0.096	0.206	0.218	0.142	0.221
	P	0.072	0.582	0.234	0.208	0.415	0.202
**Inferior temporal**	*r*	0.072	0.129	0.214	0.070	-0.177	-0.015
	P	0.681	0.459	0.217	0.688	0.309	0.930
**Central**	*r*	0.172	0.087	0.236	0.167	-0.060	0.073
	P	0.323	0.620	0.172	0.337	0.732	0.676

**B. Right Eye**		**CIRCUMPAPILLARY RETINAL NERVE FIBER LAYER**					
**VISUAL FIELD**		**Full**	**Superior nasal**	**Inferior nasal**	**Superior temporal**	**Inferior temporal**	**Macular**
**Full**	*r*	-0.034	-0.168	0.022	0.171	-0.081	-0.014
	P	0.842	0.329	0.897	0.319	0.637	0.937
**Superior nasal**	*r*	0.033	-0.015	-0.051	0.220	-0.031	-0.030
	P	0.846	0.929	0.769	0.196	0.856	0.862
**Inferior nasal**	*r*	-0.074	-0.159	0.156	0.031	-0.146	-0.080
	P	0.669	0.354	0.364	0.858	0.395	0.641
**Superior temporal**	*r*	-0.078	-0.160	0.027	0.085	-0.119	-0.033
	P	0.651	0.352	0.874	0.620	0.490	0.849
**Inferior temporal**	*r*	-0.096	-0.284	0.011	0.143	-0.120	0.049
	P	0.576	0.093	0.951	0.407	0.485	0.779
**Central**	*r*	0.053	-0.099	-0.021	0.203	0.024	0.097
	P	0.759	0.565	0.904	0.235	0.888	0.574
