# Supplementary material for: Visual Field Deficits in Albinism in Comparison to Idiopathic Infantile Nystagmus
Source: Invest Ophthalmol Vis Sci. 2024 Feb 6;65(2):13. doi: 10.1167/iovs.65.2.13 (PMC10854418; doi:10.1167/iovs.65.2.13)
Supplement: Supplement 3 [file iovs-65-2-13_s003.pdf]

Supplementary Table 3: *The table shows that there were no significant correlations between the naso-temporal asymmetry of the ganglion cell complex (GCC: ganglion cell layer + inner plexiform layer) in PwA and visual field measurements.*

The GCCT-I-Quotient and GCCT-II-Quotient are defined as mean GCC thickness in t I / n I and t II / n II, respectively, as shown in ***Figure 1D****. GCCT-II-Quotient could not be determined on 2 participants because of clipping.*

		Visual Field Measure	
OCT measure		**foveal**	**T-N**
GCCT-I-Quotient	*r*	0.120	0.222
	P	0.412	0.125
GCCT-II-Quotient	*r*	0.130	0.162
	P	0.383	0.276
